# Cancer therapy resistance converges on immune evasion: a unified framework from immunotherapy to targeted, endocrine, and DNA repair pathways

**DOI:** 10.1186/s13046-026-03798-0

**Published:** 2026-07-31

**Authors:** Ha-Eun Jeong, Heesun Choi, Wan-Teck Lim, Su Bin Lim, Joon-Yong An

**Affiliations:** 1 Department of Integrated Biomedical and Life Science, Korea University, Seoul, 02841 Republic of Korea; 2 National Research Laboratory for Convergence Degradation Biology, Korea University, Seoul, 02841 Republic of Korea; 3 School of Biosystem and Biomedical Science, College of Health Science, Korea University, Seoul, 02841 Republic of Korea; 4 Division of Medical Oncology, National Cancer Centre Singapore, Singapore, 168583 Singapore; 5 Department of Biochemistry & Molecular Biology, Ajou University School of Medicine, Suwon, 16499 Republic of Korea; 6 BK21 R&E initiative for Advanced Precision Medicine, Department of Biomedical Sciences, Graduate School of Ajou University, Suwon, 16499 Republic of Korea; 7 L-HOPE Program for Community-Based Total Learning Health Systems, Korea University, Seoul, 02841 Republic of Korea

**Keywords:** Cancer therapy resistance, Immune evasion, Tumor microenvironment, Targeted therapy, PARP inhibitor resistance, Immunotherapy, Combination therapy

## Abstract

**Background:**

Cancer immunotherapy, including immune checkpoint inhibitors, chimeric antigen receptor T-cell (CAR-T) therapy, and bispecific antibodies, has transformed the treatment of advanced malignancies, yet primary and acquired resistance remain major barriers to durable benefit. Across these therapies, resistance reflects the failure of one or more of three shared requirements for effective antitumor immunity: tumor immune visibility, lost through defective antigen presentation and interferon signaling; immune access to tumor nests, restricted by stromal and vascular barriers; and effector cell function, undermined by suppressive myeloid and regulatory populations, metabolic restriction, and loss of tertiary lymphoid structures.

**Main body:**

In this review, we argue that resistance to targeted therapies, endocrine therapies, and PARP inhibitors should not be treated as a problem separate from immunotherapy resistance. Rather, these resistant states often converge on the same three axes that govern immunotherapy resistance (immune visibility, access, and effector function), yielding a unified framework across therapeutic classes. Using canonical immunotherapy resistance as a reference model, we examine how osimertinib resistance, androgen receptor (AR)-pathway-resistant states, and BRCA reversion-mediated PARP inhibitor resistance may remodel these same immune-evasion axes. Targeted therapy resistance provides the most complete case, with epithelial-mesenchymal transition, PD-L1 upregulation, STING suppression, and immune exclusion extending across all three axes. AR pathway reactivation shows plausible links to immune remodeling through direct MHC-I repression and myeloid reprogramming, although the clinical evidence base remains less mature. BRCA reversion illustrates a mechanistically suggestive dual-valence state in which restored DNA repair may diminish innate immune sensing while also generating computationally predicted junctional neopeptides whose immunogenicity remains to be validated.

**Conclusions:**

By reframing resistance as a new immune state that converges on the same three immune-evasion axes rather than a terminal event, this review shifts attention from why a therapy failed to which immune-evasion axis now predominates and how it might be countered. This perspective supports mechanism-matched combination therapy, adaptive molecular monitoring, and cautious exploitation of vulnerabilities generated by the resistant state itself. As an organizing model rather than a claim of uniform causality, the framework now requires prospective validation.

## Background

Over the past two decades, oncology has shifted from broadly cytotoxic therapy toward precision medicine and immunotherapy. This transition has made clear that durable cancer control depends not only on direct tumor killing, but also on whether malignant cells remain visible to the immune system and accessible to immune effectors competent to eliminate them. Across therapeutic modalities, durable benefit is therefore shaped by a smaller set of shared immune requirements.

Most discussions of therapy resistance, however, still separate immunotherapy resistance from resistance to targeted, endocrine, or DNA repair-directed therapies. In practice, this distinction often obscures the biological overlap between these states. Resistance to any of these modalities can remodel antigen presentation, alter inflammatory signaling, reshape stromal architecture, and change the composition and functional state of the tumor microenvironment (TME). The tumor that emerges after one therapy may therefore already be conditioned either for response or for resistance to the next immune-based intervention.

We therefore propose a convergence model in which distinct resistance mechanisms are interpreted through three shared requirements of effective antitumor immunity. Throughout this review these are framed as visibility, access, and effector function, reflecting whether tumor cells can be recognized, reached, and eliminated. This framework organizes canonical resistance mechanisms in immunotherapy and clarifies how resistance states arising after targeted therapy, endocrine therapy, and PARP inhibition can converge on the same immune-evasion axes.

In this review, resistance refers not simply to pharmacologic escape from a drug, but to the broader biological state that emerges after therapy, including changes in antigenicity, stromal architecture, and immune composition. Likewise, convergence refers to the observation that post-resistance states can share immune-evasive features, whether these arise as direct consequences of resistance mechanisms, as parallel effects of shared upstream programs, or as persistent conditions carried through therapeutic escape. The framework is therefore intended not as a claim of uniform causality in every setting, but as a practical way to interpret the immune state of the resistant tumor and to guide mechanism-matched intervention.

## Therapeutic modalities and the shared requirements for durable antitumor immunity

**Fig. 1:**
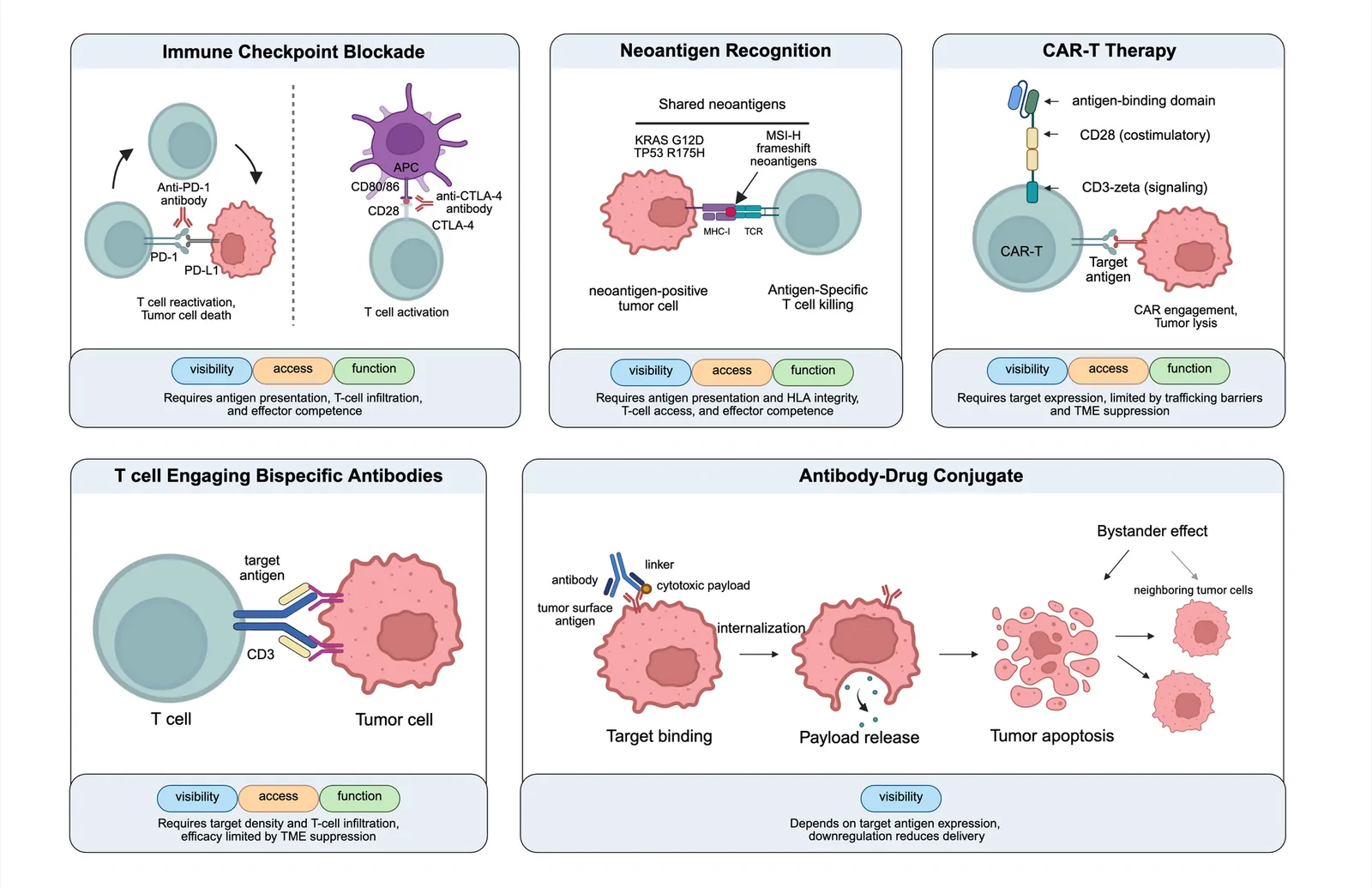
Mechanism-matched therapeutic modalities within the convergence framework. Immune checkpoint blockade, neoantigen recognition, CAR-T cell therapy, T-cell-engaging bispecific antibodies, and antibody-drug conjugates are positioned according to the immune-evasion axis or axes most relevant to their activity. Immune checkpoint blockade depends on preserved antigen presentation, T-cell infiltration, and effector competence. Neoantigen recognition depends on immune visibility through MHC-I-dependent presentation of shared neoantigens (including hotspot mutations and MSI-H frameshift neoantigens), as well as on immune access and effector function. CAR-T cell therapy bypasses peptide-HLA restriction but remains limited by target-antigen expression, trafficking barriers, and suppressive tumor microenvironments. T-cell-engaging bispecific antibodies redirect T cells to tumor cells but also require adequate access and local effector function. Antibody-drug conjugates depend on maintained tumor-surface target expression for effective payload delivery. Colored labels indicate the dominant immune-evasion axis or axes associated with each modality; all modalities ultimately depend on visibility, access, and effector function, and only the primary limiting axis or axes are highlighted here. Small-molecule targeted therapies, whose activity depends on oncogene inhibition rather than directly on these immune-evasion axes, enter the framework through their resistance states and are therefore detailed in Figs. 3 and 4 rather than shown here

Cancer therapies that intersect with antitumor immunity are structurally diverse, but their durable efficacy depends on a smaller set of shared biological requirements. A useful distinction is between canonical immunotherapies, such as immune checkpoint blockade, CAR-T cell therapy, and T-cell-engaging bispecific antibodies, and therapeutic platforms with major immunologic consequences, including antibody-drug conjugates and small-molecule targeted therapies. Across these modalities, resistance can be understood as failure of one or more of the three requirements introduced above: visibility, access, and effector function (Fig. 1). Grounded in the tumor immune phenotypes of the cancer-immunity cycle [1, 2], these three axes serve as the organizing framework for the remainder of the review. The modalities discussed below are introduced as reference systems that expose the same liabilities later revisited in resistant states.

Immune checkpoint blockade provides the clearest reference model because its efficacy depends on intact antigen presentation and interferon responsiveness, along with pre-existing or inducible T-cell infiltration. Loss of these features produces familiar resistance states, including impaired MHC-I antigen presentation [3], defective IFN-γ signaling [4, 5], and immune-cold tumor programs. These canonical mechanisms define the patterns that later reappear in resistant states initially selected by targeted, endocrine, or DNA repair-directed therapies.

Neoantigen-directed strategies fit within the same framework because antigen-specific elimination requires recognizable peptide-HLA targets and the downstream immune context needed for T-cell access and function. Thus, neoantigen recognition is shown on all three axes in Fig. 1, because therapeutic activity depends not only on antigen presentation but also on immune-cell access to tumor nests and preserved effector competence [1, 2].

The subsections that follow examine how other therapeutic modalities, including CAR-T cells, bispecific antibodies, antibody-drug conjugates, and small-molecule targeted therapies, expose these same liabilities from distinct starting points.

### CAR-T cell therapy highlights antigen escape as loss of immune visibility

CAR-T cell therapy bypasses MHC restriction, but it still depends on durable target visibility and a permissive microenvironment. Its clinical success in hematologic malignancies shows the efficacy of direct antigen recognition, whereas the more limited activity seen in solid tumors reveals the importance of trafficking, persistence, metabolic fitness, and resistance to suppressive cytokine signals [6]. Relapse through antigen loss, lineage switching, poor trafficking, or exhaustion shows that even highly engineered immune therapies ultimately fail through recurring deficits in visibility and effector competence [6–9].

### Bispecific antibodies expose the requirement for target density and T-cell competence

Bispecific antibodies are a broad class, but the subgroup most relevant to this section is T-cell-engaging (TCE) bispecific antibodies, which link CD3 on T cells to a tumor-associated surface antigen. This physical bridging brings T cells into close proximity with tumor cells and can induce cytotoxicity independently of T-cell receptor (TCR)-MHC interactions. TCEs have achieved clear clinical success in hematologic malignancies, with approved agents such as blinatumomab, teclistamab, and elranatamab [10–12]. Other CD3-engaging TCEs, including tarlatamab in small-cell lung cancer, further extend this approach to solid tumors [13, 14]. By contrast, biparatopic antibodies such as zanidatamab, discussed later as therapeutic counter-strategies, should be distinguished from TCEs because their principal purpose is enhanced target engagement rather than direct T-cell redirection [15, 16]. In framework terms, TCEs are especially informative because they reveal how quantitative loss of target density and impaired endogenous T-cell competence can jointly limit response.

TCE efficacy in solid tumors remains constrained by both target availability and endogenous T-cell fitness. Appropriate target antigen expression is required to form productive immune synapses, and heterogeneous target expression leaves antigen-low or antigen-negative subclones available for outgrowth under therapeutic pressure [17]. Transcriptional state changes can further reduce surface target abundance without requiring structural mutation [17].

### Antibody-drug conjugates link target recognition to immune remodeling

Antibody-drug conjugates (ADCs) combine tumor-selective antibody binding with potent chemical payloads and function as therapeutic platforms with important immunologic consequences rather than as purely cytotoxic delivery systems. Trastuzumab deruxtecan (T-DXd), for example, has expanded the therapeutic scope for HER2-low and HER2-ultralow breast cancer, first after prior chemotherapy in DESTINY-Breast04 and subsequently after endocrine therapy in the phase 3 DESTINY-Breast06 trial [18, 19]. Resistance to T-DXd can involve HER2 downregulation and impaired target binding, thereby reducing effective payload delivery [20]. These mechanisms do not simply blunt ADC efficacy; they also reshape antigen visibility and local inflammatory context, making ADC resistance relevant to subsequent immunotherapeutic combinations. ADCs are not classical immunotherapies, yet they clearly intersect with immune visibility and microenvironmental permissiveness, which places them in an important middle ground between classical cytotoxics and immunotherapies.

### Targeted therapies generate resistant immune states rather than simply losing drug sensitivity

Small-molecule targeted therapies are central to the convergence argument because they are usually discussed outside the immunotherapy literature. Yet resistance to kinase inhibition is not merely pharmacologic escape from a drug. The resistant state often includes bypass signaling, altered cytokine secretion, and perturbation of innate and myeloid immune programs, which reshape the immune conditions under which the next therapy will operate [21, 22]. These platforms therefore supply the clearest bridge between conventional drug resistance and the immune-evasion framework developed in the sections that follow.

## Canonical resistance programs that define the convergence model

**Fig. 2:**
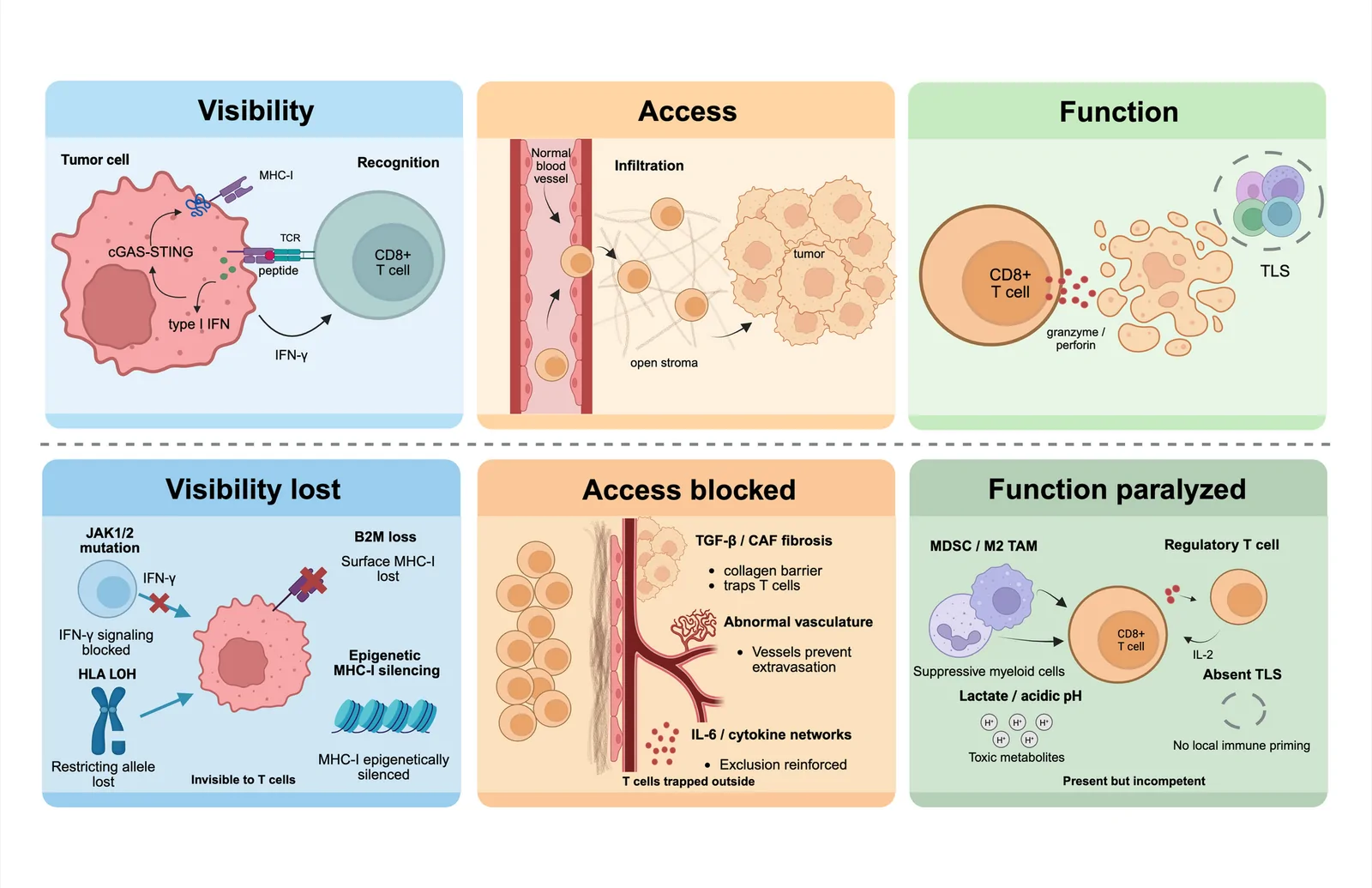
The three immune-evasion axes and representative failure states. The top row depicts the conditions that support effective antitumor immunity across the three axes of immune visibility, immune access, and effector function. Immune visibility is represented by intact antigen presentation, innate sensing, and interferon signaling that enable tumor recognition by CD8-positive T cells. Immune access is represented by T-cell trafficking through blood vessels and permissive stroma into tumor tissue. Effector function is represented by cytotoxic T-cell activity within an immune-supportive microenvironment associated with tertiary lymphoid structures. The bottom row depicts representative resistant states affecting each axis. Visibility loss includes JAK1/2 mutation, B2M loss, HLA loss of heterozygosity, and epigenetic MHC-I silencing. Access restriction includes stromal fibrosis, abnormal vasculature, and cytokine-mediated exclusion. Effector dysfunction is represented by suppressive myeloid and regulatory cell states, loss of tertiary lymphoid structures, and metabolically hostile lactate-rich acidic conditions

Before considering resistance to targeted, endocrine, and DNA repair-directed therapies, it is useful to define the reference mechanisms best established in immunotherapy. These mechanisms show how loss of immune visibility, restricted immune access, and progressive effector dysfunction combine to determine resistance, and they provide the template against which the immune consequences of these resistance states can be interpreted (Fig. 2).

### Loss of immune visibility through antigen presentation and interferon signaling defects

T-cell-mediated tumor elimination requires efficient processing and presentation of tumor antigens on MHC-I molecules. Defects in this machinery are a major cause of intrinsic resistance. Immune-evasion alterations in microsatellite instability-high (MSI-H) tumors frequently involve the antigen-presentation machinery and related immune-selection pathways [3]. Loss-of-function mutations in Janus kinase 1 and 2 (JAK1/2) can render tumor cells insensitive to IFN-γ signaling, prevent adaptive upregulation of PD-L1, and contribute to both acquired and primary resistance to PD-1 blockade [4, 5]. Likewise, loss-of-function alterations in β2-microglobulin (B2M) abolish cell-surface MHC-I expression and impair CD8-positive T-cell recognition, a mechanism implicated in acquired resistance to checkpoint blockade in lung cancer [23]. In MSI-H colorectal tumors, these defects include recurrent B2M mutations arising under immune-selective pressure [3].

Allele-specific HLA loss of heterozygosity (HLA LOH) is a key immune escape mechanism. HLA LOH occurs in approximately 40% of non-small cell lung cancers (NSCLCs) and frequently arises subclonally under immune-selective pressure [24]. HLA LOH is associated with high subclonal neoantigen burden, suggesting that tumors selectively lose the HLA allele responsible for presenting immunogenic neoantigens [24]. This has important implications for therapies targeting shared neoantigens. In a patient treated with HLA-C*08:02-restricted T cells targeting KRAS G12D, a single progressing lesion was found to have lost the chromosome 6 haplotype encoding HLA-C*08:02, providing a direct mechanism of immune evasion [25]. This pattern shows how HLA integrity constrains neoantigen-targeting therapies. Even when the tumor retains the targeted mutation, loss of the restricting HLA allele under immune-selective pressure can render the therapy ineffective.

Additional mechanisms of antigen presentation suppression have been identified. The deubiquitinase USP22 was shown to drive tumor immune evasion and checkpoint blockade resistance by stabilizing the methyltransferase EZH2, which in turn epigenetically silences MHC-I gene expression. Both genetic and pharmacologic USP22 inhibition restored MHC-I surface expression, enhanced CD8-positive T-cell killing, and overcame anti-PD-1 resistance in preclinical models [26]. Independently, large chromosome 9p21.3 deletions have been associated with an immune-cold microenvironment and primary resistance to immune checkpoint therapy across multiple cancer types. Mechanistically, loss of interferon epsilon within this deleted region has been identified as a key mediator of the resulting chemokine deficit, impairing endogenous CXCL9/10 induction and thereby limiting T-cell recruitment to the tumor [27].

Within mismatch repair-deficient (dMMR)/MSI-H tumors, response can differ according to the underlying molecular context. In endometrial cancer, MLH1-hypermethylated tumors show a colder immune phenotype and trend toward worse outcomes with immune checkpoint blockade than Lynch-like tumors in retrospective analyses [28]. Even in gastric cancer, where MSI-H status is usually favorable, molecular heterogeneity within MSI-H disease can still identify subgroups with more suppressive immune programs and worse outcomes [29].

### Immune access restriction by stromal and vascular barriers

Immune access depends on whether effectors can physically reach tumor cells. Stromal remodeling is the primary determinant. TGF-β-driven fibrosis, aberrant extracellular matrix deposition, abnormal vasculature, and cancer-associated fibroblast (CAF)-mediated immune exclusion create a spatially segregated ecosystem in which immune cells may be present but ineffective [30]. In urothelial carcinoma, TGF-β signaling in CAFs drives collagen deposition that traps T cells in the peritumoral stroma and prevents direct contact with tumor cells, providing a defining example of immune exclusion [30]. Cytokine networks such as IL-6 and related inflammatory programs reinforce this excluded state. Elevated serum IL-6 has been linked to poor outcomes in melanoma patients receiving immune checkpoint inhibitors [31]. Single-cell studies further show that CAF populations are functionally heterogeneous, with inflammatory and myofibroblastic states contributing differently to immune suppression and matrix remodeling [32].

Abnormal tumor vasculature independently constrains immune access, and vessel architecture itself can be actively remodeled. In murine breast cancer and pancreatic neuroendocrine tumor models, combined anti-vascular endothelial growth factor receptor 2 (VEGFR2) and anti-PD-L1 treatment induced high endothelial venule (HEV)-like neovessels within tumors, and these HEV-bearing regions showed a three- to tenfold enrichment of intratumoral T and B lymphocytes in their periphery [33]. This mechanistic link between vascular normalization and lymphocyte access establishes the preclinical rationale for anti-VEGF-based combination strategies later considered as access-directed interventions.

### Effector dysfunction from suppressive cells, metabolic hostility, and immune architecture

Effector function depends on whether immune cells that reach the tumor can remain active and competent. MDSCs, regulatory T cells, and M2-polarized macrophages are recurrent effectors of immune resistance because they blunt cytotoxic T-cell activity regardless of the therapy that first selected the resistant state. In preclinical models, colony-stimulating factor 1 (CSF1)/CSF1 receptor (CSF1R) signaling can recruit and sustain immunosuppressive tumor-associated macrophages (TAMs), establishing a negative feedback loop that limits checkpoint efficacy [34]. Regulatory T cells further consolidate suppression by competing for IL-2 and secreting TGF-β and IL-10, while myeloid-derived suppressor cells (MDSCs) deplete arginine and cysteine and produce reactive oxygen species, collectively creating a metabolic and signaling environment hostile to effector T-cell function [17, 35–37]. More broadly, the same TME cues that exhaust tumor-reactive T cells can also impair CAR-T cells or T cells redirected by TCEs, showing that effector dysfunction is a shared endpoint across otherwise different therapeutic modalities [17, 35–37].

The absence of tertiary lymphoid structures (TLS) further compounds effector dysfunction by removing a source of local immune priming. TLS presence and maturation are recognized as positive determinants of checkpoint response independently of PD-L1 expression [38–41]. Metabolic deregulation adds a further layer because lactate accumulation acidifies the microenvironment and directly impairs T-cell proliferation [42]. Beyond the local microenvironment, systemic mediators such as GDF-15, tumor-derived PD-L1-bearing extracellular vesicles, and intestinal dysbiosis extend effector dysfunction to the host level, illustrating that the effector axis can be compromised beyond the tumor boundary [43–46]. Effector failure is therefore sustained by local cellular suppression as well as metabolic and host-level signals.

Certain oncogenic drivers and genetic states actively orchestrate an immunosuppressive microenvironment and thereby predispose tumors to primary resistance. In KRAS-mutant NSCLC, co-occurring alterations in STK11 (also known as LKB1) are strongly associated with primary resistance to PD-1 blockade [47]. STK11 loss promotes an immune-cold state characterized by reduced CD8-positive T-cell infiltration and low PD-L1 expression. Exploratory analyses suggest that STK11- and KEAP1-altered tumors respond poorly to PD-(L)1-based therapy, and that adding CTLA-4 blockade to PD-(L)1 blockade plus chemotherapy may improve outcomes (median overall survival 15.8 vs. 7.3 months; hazard ratio 0.64, 95% confidence interval 0.40–1.04 vs. PD-(L)1 blockade plus chemotherapy); however, these subgroups are small, the benefit did not reach statistical significance, and outcomes remain inferior to those in wild-type tumors [48, 49]. Alterations in DNA repair pathways, beyond causing MSI-H, can likewise shape intrinsic immunogenicity and resistance. Across tumor types, low tumor mutational burden and absence of a T-cell-inflamed gene-expression profile remain recurring correlates of primary non-response [50].

## Convergence of targeted, endocrine, and DNA repair-directed therapy resistance on immune evasion

**Fig. 3:**
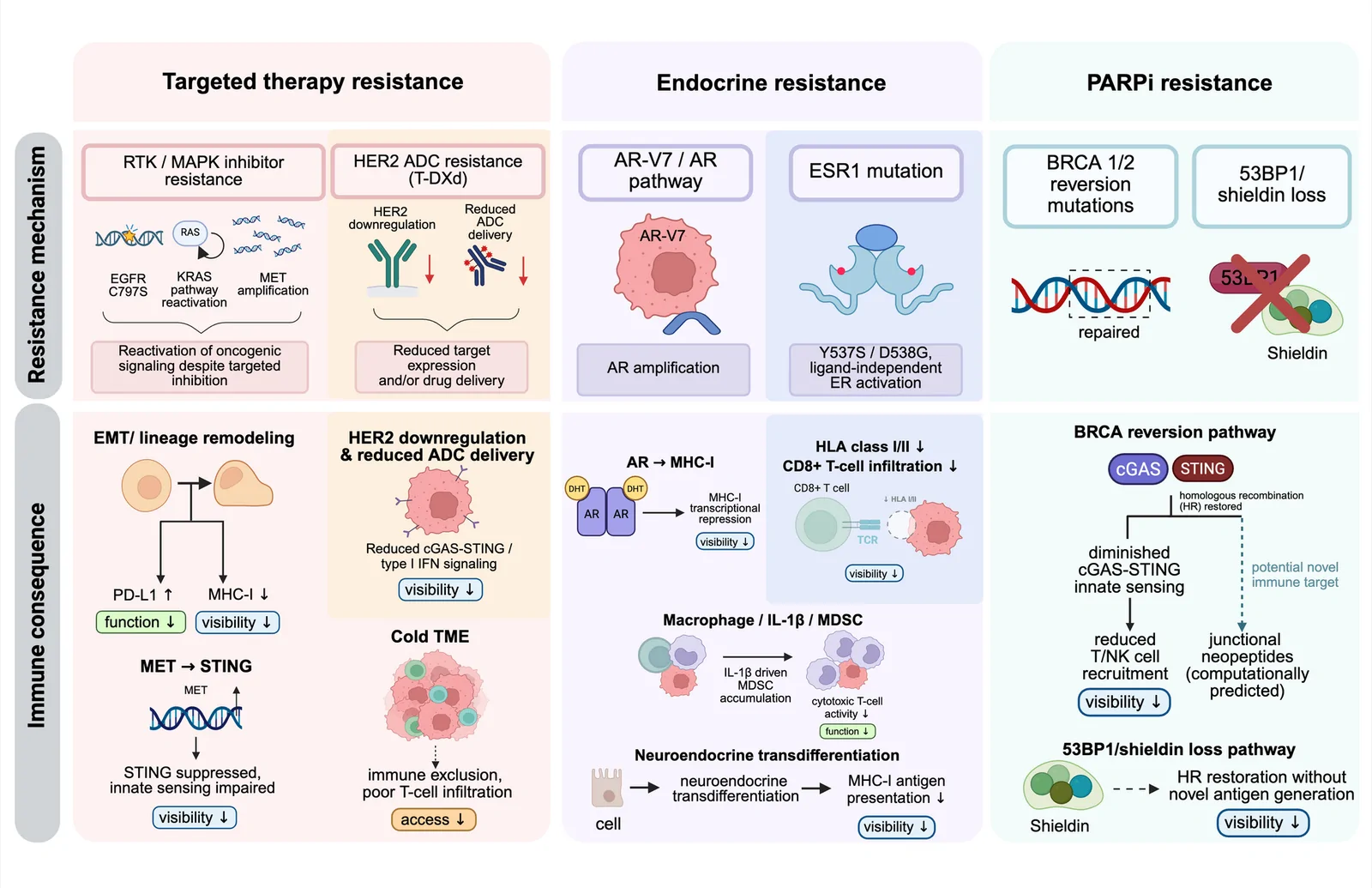
Representative resistance states after targeted, endocrine, and DNA repair-directed therapy. The top row shows resistance mechanisms in each therapy class, and the bottom row shows the corresponding immune-remodeling consequences along the three axes of visibility, access, and effector function. In targeted therapy resistance (left column), RTK/MAPK inhibitor resistance (EGFR C797S, KRAS pathway reactivation, and MET amplification) drives EMT and lineage remodeling with reduced MHC-I and elevated PD-L1, MET-driven STING suppression with impaired innate sensing, and a cold, immune-excluded microenvironment. Resistance to the HER2 ADC T-DXd, arising from HER2 downregulation and reduced ADC delivery, lowers cGAS-STING and type I interferon signaling. In endocrine resistance (middle column), AR-V7, AR-pathway persistence, and AR amplification act through AR-mediated repression of MHC-I, IL-1β-driven MDSC accumulation with reduced cytotoxic T-cell activity, and neuroendocrine transdifferentiation with loss of antigen presentation. ESR1-mutant ER-positive breast cancer (Y537S/D538G) is a secondary route in which reduced HLA class I and II and lower CD8-positive T-cell infiltration diminish visibility. In PARPi resistance (right column), two routes restore homologous recombination: BRCA1/2 reversions lower cGAS-STING sensing and T- and NK-cell recruitment, while 53BP1/shieldin loss restores homologous recombination through BRCA1-independent end resection without generating new junction-derived antigens. Computationally predicted junctional neopeptides (unvalidated immunogenicity) form a separate dashed branch, flagged as a novel immune target rather than an evasion consequence. Solid arrows denote well-established mechanistic or clinical links; dashed arrows denote hypothetical, emerging, preclinical, or correlative ones. ADC, antibody-drug conjugate; AR, androgen receptor; cGAS, cyclic GMP-AMP synthase; DHT, dihydrotestosterone; EMT, epithelial-mesenchymal transition; ESR1, estrogen receptor 1; HLA, human leukocyte antigen; HR, homologous recombination; IFN, interferon; IL-1β, interleukin-1β; MAPK, mitogen-activated protein kinase; MDSC, myeloid-derived suppressor cell; MET, MET proto-oncogene receptor tyrosine kinase; MHC, major histocompatibility complex; NK, natural killer; RTK, receptor tyrosine kinase; STING, stimulator of interferon genes; T-DXd, trastuzumab deruxtecan; TME, tumor microenvironment

The resistant states selected by targeted therapies, endocrine therapies, and PARP inhibitors (PARPi) can be read through the same three axes: immune visibility, immune access, and effector function. What matters is therefore not only how each resistance mechanism restores tumor growth, but how it reshapes the tumor-immune interface encountered by subsequent therapy (Fig. 3).

### Targeted therapy resistance remodels immune visibility, access, and function

Resistance to epidermal growth factor receptor (EGFR) tyrosine kinase inhibitors (TKIs) classically arises on-target through acquired mutations such as EGFR C797S, in which substitution of the cysteine-797 residue eliminates the covalent anchor required for osimertinib binding [51]. Beyond such pharmacologic escape, however, the resistant state also remodels the tumor-immune interface. Among non-genetic resistance routes, epithelial-mesenchymal transition (EMT) is a recurrent feature of EGFR TKI tolerance and acquired osimertinib resistance, with classical genotypic and histologic studies documenting EMT in NSCLC tumors that have escaped EGFR inhibition [52]. EMT-associated transcriptional programs in lung adenocarcinoma further co-occur with elevated PD-L1 and multiple inhibitory immune checkpoints alongside an inflammatory, immunosuppressive microenvironment [53]; functionally, the EMT regulator ZEB1 represses miR-200 to upregulate PD-L1 and impair CD8-positive T-cell effector function [54].

KRAS G12C-directed therapy offers a second instance in which resistance mechanisms intersect with the immune state at multiple timepoints. Shortly after G12C inhibitor exposure, cancer cells exhibit non-uniform adaptive responses in which newly synthesized KRAS(G12C) is sustained in its drug-insensitive active state through EGFR and Aurora kinase signaling, enabling a subset of cells to bypass inhibition and resume proliferation [55]. In a single-patient multi-region study of paired pre- and post-sotorasib biopsies, whole-exome and RNA sequencing revealed ecosystem-level reshaping of the TME, including KRAS pathway reactivation, EMT, metabolic reprogramming, and reduction of immune gene signatures consistent with an immunologically cold state [56, 57]. Co-occurring STK11 or KEAP1 alterations further entrench a cold and suppressive state by simultaneously weakening the visibility and effector-function axes [47, 48]. KRAS G12C resistance therefore spans the visibility and effector-function axes through a trajectory that differs molecularly from EGFR-driven resistance but produces analogous multi-axis immune consequences.

For HER2-directed ADCs (grouped here with targeted therapy as target-directed agents, consistent with their middle-ground role introduced in Section "Antibody-drug conjugates link target recognition to immune remodeling"), the immune consequences map most directly onto visibility. T-DXd pairs HER2 binding with intratumoral delivery of a topoisomerase I inhibitor payload (DXd) that itself induces DNA damage, cyclic GMP-AMP synthase-stimulator of interferon genes (cGAS-STING) activation, and a type I interferon response, enhancing dendritic-cell activation and CD8-positive T-cell-mediated cytotoxicity in HER2-expressing preclinical models [58]. Resistance through HER2 downregulation therefore reduces both effective payload delivery and the immunostimulatory signal that would otherwise support adaptive antitumor responses, producing a combined pharmacologic and visibility deficit. How this resistant state further affects effector function, and what it implies for subsequent HER2-directed immunotherapy, remains to be established in patients.

Across these targeted-therapy contexts, several clinical observations reinforce the shared framework. MET amplification has been linked to suppression of STING-driven tumor immunogenicity, reduced CD8-positive T-cell and natural killer (NK)-cell infiltration, and a dysfunctional immune milieu [59]. In KRAS-mutant tumors, allele-specific effects also matter: G12D mutations are associated with lower PD-L1 expression, reduced T-cell infiltration, and poorer outcomes to PD-(L)1 blockade than non-G12D variants [60]. EGFR-mutant tumors also typically have lower tumor mutational burden than smoking-associated NSCLC [61]. Consistent with this, prospective dual checkpoint blockade after TKI resistance has shown poor activity [62]. Once a cold and suppressive TME has been established, checkpoint intensification alone is often insufficient. The partial benefit seen with anti-angiogenic combinations in IMpower150 and in subsequent EGFR TKI-resistant cohorts is consistent with the view that vascular normalization may help reopen immune access in this setting [63, 64]. The strength of this evidence makes targeted therapy resistance a natural anchor for the convergence argument; the following sections examine whether analogous immune remodeling occurs in settings where the mechanistic data are less mature.

### AR pathway resistance as a test case for lineage-immune coupling

Androgen receptor (AR) pathway resistance provides a further test case for this framework because it offers a more direct, albeit incompletely resolved, link between lineage signaling and immune remodeling. In prostate cancer, resistance to AR signaling inhibitors is driven by amplification, point mutation, and splice variation [65, 66]. These mechanisms can preserve or reactivate AR signaling while broader AR-pathway remodeling can reshape immune effector function and myeloid-state biology [67], not merely preserve lineage survival.

AR-V7 is especially instructive because it marks a resistant lineage state that is clinically aggressive and potentially immunologically relevant. In castration-resistant prostate cancer (CRPC), specific ligand-binding domain substitutions can even convert AR antagonists into agonists [65, 66]. Yet the current evidence should be interpreted cautiously. AR-V7 is strongly associated with resistance to enzalutamide and abiraterone, and small non-randomized immunotherapy studies suggest limited checkpoint activity in this setting [68–70]. Direct evidence that AR-V7 itself establishes an immune-cold microenvironment remains limited. The broader AR-pathway-resistant state, however, shows several plausible links to immune remodeling. A genome-wide CRISPR-interference (CRISPRi) screen identified AR as a direct transcriptional repressor of MHC-I antigen-presentation genes, and AR inhibition with enzalutamide enhanced CD8-positive T-cell recognition in preclinical models and was associated with increased surface MHC-I expression in paired patient tissues [71]. This immune benefit eroded as castration resistance developed, coinciding with glucocorticoid receptor upregulation that re-suppressed MHC-I [71]. In a complementary model, ectopic AR-V7 expression in prostate cancer cells was associated with expansion of FoxP3-positive regulatory T cells and an immunosuppressive phenotype [72].

At the level of the TME, enzalutamide-resistant prostate cancer cells have been shown to upregulate PD-L1, suppress chemotaxis and IFN-response pathways, promote MDSC differentiation, and skew macrophages toward an M2 phenotype [67]. Within CRPC, granulocytic MDSCs are the predominant myeloid subset and correlate with inferior survival [73]. Mechanistically, IL-23 secreted by tumor-infiltrating myeloid cells can directly activate AR signaling in prostate cancer cells under androgen-deprived conditions, establishing a feedforward loop between myeloid inflammation and castration resistance [74]. This myeloid dependency is therapeutically actionable: C-X-C motif chemokine receptor 2 (CXCR2) inhibition combined with enzalutamide reduced intratumoral myeloid infiltration and produced durable clinical responses in a subset of patients with metastatic CRPC, identifying myeloid chemotaxis as a targetable node within the effector-function axis [75]. Supporting this broader link, androgen-responsive macrophage programs have been shown to drive IL-1β production and MDSC accumulation after androgen deprivation, consistent with an impaired cytotoxic effector state [76].

Beyond effector-function remodeling, AR pathway resistance can also compromise immune visibility through lineage plasticity. Neuroendocrine transdifferentiation is driven by transcription factors such as ASCL1 and extinguishes the AR program [77, 78]. This transition is accompanied by reduced MHC-I antigen presentation, rendering transdifferentiated tumors poorly visible to CD8-positive T cells [79]. This lineage switch therefore represents a convergence point where resistance to AR-directed therapy simultaneously generates both effector-function deficits through myeloid remodeling and visibility deficits through antigen-presentation loss.

Estrogen receptor (ER)-positive breast cancer offers a briefer and less-resolved endocrine comparison. In that setting, ESR1 ligand-binding domain mutations such as Y537S and D538G confer constitutive, ligand-independent ER activation and drive resistance to aromatase inhibitors [80]. Emerging evidence suggests that ESR1-mutant metastatic tumors show downregulation of HLA class I and class II molecules and reduced CD8-positive T-cell infiltration relative to ESR1 wild-type disease [81], raising the possibility that endocrine resistance in this setting may intersect with the visibility axis. Separately, cyclin-dependent kinase 4/6 (CDK4/6) inhibitor treatment can induce senescence with a senescence-associated secretory phenotype that remodels immune composition and carries both immunostimulatory and immunosuppressive potential depending on context [82]. The evidence here remains preliminary, and ER-positive breast cancer is generally considered immunologically cold irrespective of endocrine resistance status, making it a secondary comparison rather than the central endocrine example.

### DNA repair restoration and PARPi resistance create both immune loss and immune opportunity

PARPi are molecularly targeted agents whose resistance centers on the restoration of DNA repair. Within this framework, PARPi resistance bears most directly on immune visibility. BRCA1/2 reversion mutations restore functional homologous recombination (HR) and reverse one of the liabilities that initially made the tumor therapeutically vulnerable [83–85]. Similar reversion-mediated resistance has been documented in pancreatic ductal adenocarcinoma with germline BRCA mutations [86]. Case studies have also shown the accumulation of multiple distinct secondary BRCA1/2 reversion alterations within a single patient [87]. Prior exposure to platinum or radiation may select for reversion-bearing clones even before PARPi treatment [88], and we expect treatment sequence to shape the later immune state as well. However, BRCA reversion is only one of several mechanisms through which PARPi resistance emerges, and the immune consequences differ across these routes. Loss of 53BP1 or its downstream effector complex shieldin restores HR in BRCA1-deficient cells [89]. Because this route restores HR through end resection rather than reverting the mutant BRCA1 allele, it does not create new reversion junction sequences; we therefore expect it to confer resistance without the junctional neopeptides that genetic reversions might otherwise expose as novel immune targets [90]. Replication fork protection, mediated by stabilized RAD51 loading and loss of fork-degrading factors, can bypass the requirement for HR entirely, producing a resistant state whose immune-visibility consequences remain largely uncharacterized [91]. At the level of drug pharmacology, PARP1 mutations that reduce inhibitor trapping [92] and ABCB1-mediated drug efflux [93] confer resistance through mechanisms that are unlikely to directly alter the tumor-immune interface. This diversity means that PARPi resistance does not map uniformly onto a single immune-evasion axis: BRCA reversion may reconfigure visibility in dual-valence fashion, 53BP1/shieldin loss may restore HR without an accompanying antigenic shift, and pharmacologic resistance may leave the pre-existing immune state largely unchanged.

Among these pathways, the reversion route is the most thoroughly characterized immunologically, and it illustrates a dual-valence reconfiguration of the visibility axis. In BRCA-deficient tumors, replication stress and PARP inhibition can generate cytoplasmic DNA fragments that activate cGAS-STING signaling, inducing type I interferon and downstream chemokines such as CXCL10 and CCL5 that recruit CD8-positive T cells to the tumor [94]. These observations suggest that BRCA reversion, by restoring HR and reducing replication stress, would attenuate this innate immune visibility. At the same time, the reversion process itself may create novel junctional neopeptides that have been computationally predicted but not yet experimentally validated as immunogenic targets [90]. Indirect clinical signals are consistent with this possibility, although they require careful interpretation. The phase 2 MOONSTONE/GOG-3032 trial of niraparib plus dostarlimab in BRCA wild-type platinum-resistant ovarian cancer was closed for futility after an objective response rate of only 7.3% [95]. However, these tumors were HR-proficient from the outset and therefore lacked the cGAS-STING-driven immune priming that characterizes BRCA-deficient disease [94]. BRCA-reverted tumors occupy a biologically distinct state. They originate from an HR-deficient, immunologically active baseline and transition to HR restoration through reversion, potentially carrying novel junction-derived antigens that are absent in constitutively HR-proficient tumors [90]. The MOONSTONE result therefore illustrates the difficulty of checkpoint addition in immune-cold, HR-proficient disease, but should not be directly extrapolated to the post-reversion setting, where the immune trajectory and therapeutic window may differ. Immunogenomic profiling of a separate PARPi-plus-immunotherapy cohort further showed that patients lacking both an HR deficiency signature and a positive immune score had zero objective responses, reinforcing the notion that immune-evasion depth determines combination benefit [96]. The implication is not that every reversion state becomes uniformly immune-cold, but that DNA repair restoration can reduce one source of immune visibility while potentially creating another antigenic state. BRCA reversion therefore acts primarily on the visibility axis, but with dual valence.

**Fig. 4:**
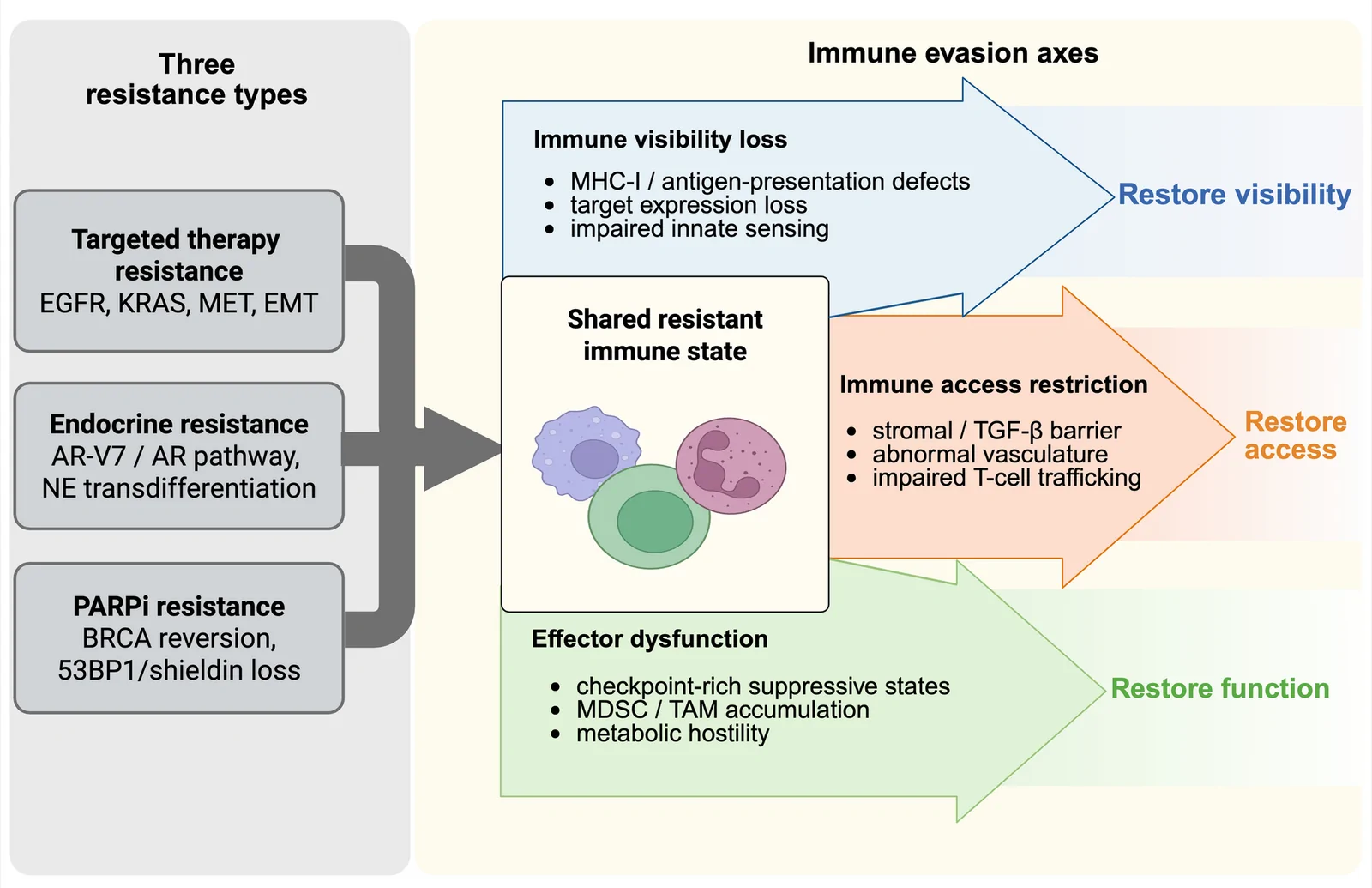
Convergence model linking targeted, endocrine, and DNA repair-directed therapy resistance to shared immune-evasion axes. Distinct resistance routes are shown on the left (EGFR/KRAS/MET-driven targeted therapy resistance with EMT and lineage remodeling; AR-V7/AR-pathway resistance with neuroendocrine transdifferentiation; and PARP inhibitor resistance via BRCA reversion or 53BP1/shieldin loss). These converge on a shared resistant immune state (center) organized around three immune-evasion axes (right): visibility loss, access restriction, and effector dysfunction. Each axis is paired with the corresponding therapeutic objective of restoring visibility, access, or function. The schematic summarizes the proposed framework for interpreting resistant tumors according to their dominant immune deficit

These three examples illustrate resistant immune remodeling at different levels of mechanistic resolution. Targeted therapy resistance provides the most complete case, with the most direct evidence for visibility and effector-function deficits, and immune exclusion implicating the access axis. Endocrine resistance identifies a plausible but less fully validated coupling between lineage signaling and immune remodeling. BRCA reversion, in turn, shows that resistant states can reconfigure immune visibility in bidirectional ways rather than simply extinguish it. Read together, these examples support the broader framework while preserving clear differences in mechanistic depth across settings (Fig. 4).

### Caveats: heterogeneity, spatial architecture, and temporal dynamics

The convergence framework treats each resistant tumor as a relatively coherent immune state, but several biological caveats warrant explicit acknowledgment before mechanism-matched intervention strategies can be operationalized. First, intratumoral heterogeneity means that distinct subclones within the same lesion can simultaneously occupy different immune-evasion axes; some clones may primarily suffer visibility loss while others harbor effector-suppressive niches, creating a mosaic immune state that is poorly captured by bulk transcriptomic or genomic profiling [24]. Second, spatial architecture matters: even where the overall TME is permissive, T-cell infiltration may be restricted to specific niches, and immunosuppressive cells such as regulatory T cells, MDSCs, and exhausted T cells often cluster in particular regions defined by stromal organization or vascular access [30]. Third, the immune state of a resistant tumor is not static: temporal dynamics during and after resistance evolution can reshape the immune microenvironment over weeks to months, with sequential shifts in immune cell composition, checkpoint expression, and antigen presentation that single-timepoint biopsies cannot resolve [97]. These caveats imply that the framework should be operationalized with longitudinal, spatially resolved, and ideally single-cell-level immune profiling rather than from snapshot bulk data alone.

## Therapeutic implications of the convergence framework

Across these cases, resistance reshapes the same three axes whatever its molecular trigger, and we now turn to what this implies for treatment. Even allowing for the heterogeneity and temporal caveats, the practical shift is from selecting therapy by drug class to repairing the dominant immune-evasion deficit in a given resistant state. In practice this requires first identifying whether visibility, access, or effector function is most limiting, then matching intervention to that specific deficit rather than escalating empirically. This places as much weight on reading the resistant state as on the repair strategies it indicates. The sections below follow this logic, from matching combination therapy to the dominant axis (Section "Matching combination therapy to the dominant immune-evasion axis") to restoring immune visibility and exploiting resistance-generated antigens (Section "Restoring immune visibility and exploiting resistance-generated antigens"), re-establishing immune access and effector function (Section "Restoring immune access and effector function"), and extending these principles through next-generation engineered antibody and cellular therapies (Section "Next-generation engineered antibody and cellular therapies").

### Matching combination therapy to the dominant immune-evasion axis

Combination therapy is the most direct application of this principle. Tumors defined primarily by effector dysfunction may be candidates for ra tional checkpoint combinations or myeloid-targeting strategies, whereas tumors that have lost visibility may be better approached with antigen-restoring or neoantigen-directed strategies. This framing also helps explain why some settings, such as STK11/KEAP1-mutant disease, may require broader immune reprogramming than PD-(L)1 blockade alone [48].

Combination therapy should therefore be understood less as empirical stacking of active drugs than as an attempt to repair the dominant deficit in immune visibility, access, or function. Beyond combinations that add CTLA-4 blockade to PD-(L)1 inhibition, inhibitory receptors such as lymphocyte activation gene 3 (LAG-3) and T-cell immunoreceptor with Ig and ITIM domains (TIGIT) have emerged as clinically relevant combination targets. Dual PD-1/LAG-3 blockade with relatlimab plus nivolumab (RELATIVITY-047) improved progression-free survival over nivolumab alone in advanced melanoma [98]. TIGIT-directed combinations have shown encouraging early signals in NSCLC but mixed phase 3 validation across thoracic malignancies [99, 100]. Additional checkpoints such as V-domain Ig suppressor of T-cell activation (VISTA) are conceptually attractive in myeloid-rich and immune-excluded states, although the clinical positioning of VISTA-directed therapy remains early [101]. The central translational question is therefore not simply which agent to add, but which biologic deficit in the resistant state is most plausibly limiting response and should be prioritized for correction (Table 1).

**Table 1:** Candidate operational priorities for mechanism-matched intervention across dominant immune-evasion axes

Dominant immune-evasion axis	Clinical or biologic clues	Resistance biomarker	Immune-state biomarker	Preferred intervention logic	Evidence level	References
Immune visibility loss	MHC-I/HLA loss; target downregulation; impaired innate sensing; reduced cGAS-STING signaling	ctDNA reversion detection; resistance mutation profiling (e.g., EGFR C797S, BRCA reversion)	HLA/MHC-I expression profiling; neoantigen prediction; target-antigen expression assessment	Restore antigenicity / targetability; consider neoantigen-directed vaccines or TCR/pHLA-directed strategies when actionable epitopes are retained or generated	Neoantigen vaccines: Phase I-II; TCR-T (KRAS G12D): case report; TCR-T (MAGE-A4): Phase II; pHLA antibodies: preclinical; epigenetic MHC-I restoration: preclinical	[26, 108, 110, 113–119]
Immune access restriction	Stromal exclusion; abnormal vasculature; low intratumoral T-cell infiltration; TGF-β/CAF-dominant architecture	On-treatment ctDNA dynamics as surrogate for treatment response	Spatial immune profiling; stromal/vascular density assessment; T-cell infiltration scoring; TLS localization	Microenvironmental remodeling: anti-VEGF-based normalization, TGF-β blockade, or stromal remodeling; often paired with checkpoint-based therapy	Anti-VEGF + ICI: Phase III, approved (RCC/NSCLC); bintrafusp alfa: Phase II-III, mixed: NSCLC and BTC not met, cervical Phase II signal of activity; stromal targeting: preclinical	[63, 102, 122–126]
Effector dysfunction	Checkpoint-rich suppressive states; MDSC/TAM accumulation; metabolic hostility; exhausted or TLS-poor immune context	AR-V7 or ESR1 mutation status as resistance indicator	Immune phenotyping; myeloid-marker assessment; TLS maturation scoring; metabolic-context profiling (lactate, hypoxia)	Reverse suppressive immune states through checkpoint-based, myeloid-directed, or metabolic strategies; engineered effectors in selected settings	Dual ICI (PD-1 + LAG-3): approved, melanoma; PD-L1 + CTLA-4 + chemotherapy: approved, NSCLC; STK11/KEAP1 subgroup: Phase III post-hoc; TIGIT + ICI: Phase II positive, Phase III not met; myeloid targeting (CSF1R): preclinical to Phase Ib; engineered CAR-T (armored, SynNotch): preclinical to Phase I	[34, 48, 49, 98–100, 121, 127–130]

*Abbreviations: BTC* Biliary tract cancer, *ICI* Immune checkpoint inhibitor, *RCC* Renal cell carcinoma

We propose that targeted therapy plus immunotherapy combinations are more likely to benefit patients when the targeted agent is actively reshaping the TME rather than after a fully consolidated resistant state has formed. Concurrent targeted-plus-checkpoint combination is clinically active in the first-line setting, as shown by the lenvatinib plus pembrolizumab renal-cell carcinoma trial (CLEAR/KEYNOTE-581) [102], although that trial did not test timing or sequence. The same reasoning applies in reverse to immune-excluded tumors, where checkpoint intensification alone tends to fall short. As noted in Section "Targeted therapy resistance remodels immune visibility, access, and function", a randomized phase 2 study of nivolumab versus nivolumab plus ipilimumab in EGFR TKI-resistant NSCLC reported an overall objective response rate of only 3.2% and was closed for futility [62]. Dual checkpoint blockade cannot readily overcome immune exclusion that requires active microenvironmental remodeling as a prerequisite.

The operational tools that support this framework should be understood as enabling technologies rather than as separate therapeutic strategies. Their role is not to define a distinct resistance class, but to improve delivery, state classification, and timing of mechanism-matched interventions. In practice, these tools include liquid-biopsy assays that detect emerging resistant clones, multimodal tissue profiling that characterizes immune state heterogeneity and could in principle support axis-based classification of resistant states [103], and artificial intelligence (AI)-assisted pathology workflows that may extend this classification to routine clinical samples [104].

### Restoring immune visibility and exploiting resistance-generated antigens

Epigenetic therapies and antigen-restoring strategies are relevant here because they address tumors that have become poorly visible to the immune system. DNA methyltransferase (DNMT) inhibition can restore expression of MHC-I genes, cancer-testis antigens, and endogenous retroviral elements, thereby triggering viral-mimicry signaling and type I interferon production [105, 106]. Histone deacetylase (HDAC) inhibition can further increase antigen presentation and alter tumor-cell and tumor-immune crosstalk [107], but these effects are context-dependent and require agent-specific interpretation. EZH2-directed approaches may extend this logic to genomically defined settings such as ARID1A-mutant tumors, where chromatin-based dependencies may be therapeutically exploitable and may intersect with immune-restoring strategies [26, 108].

These interventions are most useful when integrated with immune therapies rather than used as stand-alone salvage agents, because their main contribution may be to render resistant tumors newly interpretable by the immune system rather than directly eliminating them. In this respect, epigenetic therapies function less as generic sensitizers than as tools for restoring a specific failed requirement.

This logic also points toward neoantigen-directed intervention. Resistant tumors may lose one form of immunogenicity while gaining another, as illustrated by BRCA reversion-associated neopeptides [90] or shared frameshift neoantigens in MSI-H disease [109]. Rather than assuming that resistance uniformly reduces immune targetability, this view encourages active mapping of newly created antigenic vulnerabilities. The aim is to make resistant tumors recognizable to the immune system again.

Neoantigen-based vaccination and TCR-directed therapies become most relevant when resistance creates or unmasks antigens that can be therapeutically exploited. These approaches primarily seek to restore or redirect immune visibility, although their ultimate efficacy still depends on access and effector competence. Shared frameshift neoantigens in MSI-H disease provide one model. A frameshift peptide vaccine targeting recurrent MSI-associated mutations demonstrated immunogenicity in dMMR colorectal cancer [110]. The poly-epitope structure of these mutant reading frames makes them especially attractive vaccine targets [109]. However, HLA restriction remains an important consideration for broad deployment, even for shared neoantigens, because therapeutic relevance depends on specific neoepitope-HLA pairing. Analyses of recurrent public neoantigens suggest that systematically mapping neoepitope-HLA pairs may partially address this constraint and enable off-the-shelf therapeutic development [111, 112].

Adoptive cell therapy becomes especially attractive when the resistant state can be mapped with sufficient precision to identify conserved, targetable epitopes. KRAS G12D-specific tumor-infiltrating lymphocytes provided early clinical proof of concept in colorectal cancer [25]. This idea has since been translated to engineered TCR therapy in pancreatic cancer [113]. More broadly, the phase 2 SPEARHEAD-1 trial showed meaningful activity for affinity-enhanced TCR therapy targeting MAGE-A4 in synovial sarcoma [114], supporting the translational feasibility of TCR-based approaches in solid tumors.

Peptide-HLA (pHLA) complex-targeting antibodies further extend this principle by allowing intracellular mutation-derived antigens to be addressed through antibody-like modalities. Bispecific antibodies directed against the TP53 R175H pHLA complex can activate T cells and lyse tumor cells even at low antigen density [115], thereby offering a route to exploit resistance-associated intracellular alterations without requiring patient-specific cell manufacturing.

This field is also moving clinically, although the maturity of evidence differs across platforms. In Lynch syndrome carriers, the NOUS-209 heterologous prime-boost vaccine induced durable neoantigen-specific T-cell responses [116]. Personalized multi-adjuvant neoantigen vaccine platforms have generated broad de novo responses in melanoma [117], and individualized neoantigen mRNA therapy has shown both clinical activity and vaccine-specific T-cell responses in melanoma [118, 119]. The translational challenge is to connect these antigen-discovery pipelines to real-time monitoring so that evolving vulnerabilities are recognized while still actionable. These strategies act at the antigen level, and their success ultimately depends on whether access and effector function can be concurrently maintained.

### Restoring immune access and effector function

Restoring immune access and effector function often requires active remodeling of the TME before resistant tumors can respond to immunotherapy. This may involve depletion or reprogramming of suppressive myeloid populations, reversal of metabolic hostility, and normalization of stromal or vascular barriers that physically segregate immune cells from tumor nests.

Myeloid-targeting approaches such as CSF1R or CXCR2 blockade are attractive when suppressive macrophage and granulocytic programs dominate. The central objective is to reopen effector function rather than target macrophages as an isolated compartment [34]. Clinical translation, however, has proved challenging. Anti-CSF1R antibodies combined with checkpoint inhibitors have shown modest objective response rates in advanced solid tumors, and translational studies suggest that more intensive CSF1R blockade can be counterproductive, enriching suppressive macrophage states rather than relieving immune suppression [120, 121]. These observations indicate that myeloid reprogramming must be calibrated rather than indiscriminate, and that correcting one axis of immune evasion can destabilize another if the intervention is not appropriately tuned.

Stromal and vascular normalization, including TGF-β pathway blockade and anti-VEGF-based strategies, can be understood as access-restoring strategies. Their benefit is likely to be greatest in tumors where the dominant barrier is exclusion rather than absent antigenicity alone, a view supported by vascular-normalization theory and preclinical studies of TGF-β/PD-L1 cotargeting, with partial clinical support from anti-angiogenic combination regimens in selected settings [30, 63, 122, 123]. Bintrafusp alfa, a bifunctional fusion protein that simultaneously neutralizes TGF-β and blocks PD-L1, has been the most extensively tested agent in this class. Bintrafusp alfa showed no superiority over pembrolizumab in PD-L1-high NSCLC [124]. A phase 2/3 study in biliary tract cancer likewise did not meet its primary endpoint [125]. A signal of activity has nevertheless appeared in recurrent cervical cancer after platinum failure [126]. These mixed results suggest that TGF-β-directed access restoration is not universally effective but may be most valuable when patient selection is guided by the dominant immune-evasion axis.

### Next-generation engineered antibody and cellular therapies

Next-generation engineered therapies should be interpreted as therapeutic counterstrategies rather than as general implementation tools. Armored CAR-T cells that secrete IL-12 or IL-18 [127, 128], express dominant-negative TGF-β receptors [129], or incorporate logic-gated targeting [130] seek to preserve effector function within suppressive microenvironments.

At the antibody level, biparatopic design has emerged as an active strategy for resistant HER2 biology. By simultaneously engaging two HER2 epitopes, biparatopic antibodies are intended to enhance target engagement beyond conventional monospecific agents. Zanidatamab illustrates the clinical translation of this design logic in resistant HER2-directed therapy [15]. Together with armored CAR-T approaches, these platforms exemplify how next-generation engineering can be deployed to preserve effector function or reinforce target engagement within resistant immune contexts.

## Conclusions

Cancer therapy resistance is not merely drug escape, but a transition into a new immune state. In this review, we argue that resistant states arising after targeted therapy, endocrine therapy, and PARP inhibition can converge on the same three axes that govern immunotherapy response: loss of visibility, restriction of access, and effector dysfunction. In this sense, canonical immunotherapy resistance serves less as a separate category of failure than as a useful reference model for interpreting resistant states across therapeutic classes.

At the same time, this framework should be treated as an organizing model rather than as a claim of complete biological uniformity. Some resistant states are supported by stronger mechanistic data than others, and not every case fits neatly within a single axis. BRCA reversion, for example, illustrates that resistance may simultaneously diminish one form of immune visibility while creating another potentially actionable antigenic state. The value of the framework lies in clarifying dominant immune-evasion axes, not in implying that every resistant tumor follows the same trajectory. As a narrative synthesis, this review is necessarily selective: it draws primarily on NSCLC, breast, and prostate cancer paradigms, and its conclusions may not extend uniformly to tumor types with distinct immune biology such as glioblastoma or pancreatic adenocarcinoma. Several of the proposed connections also rest on preclinical or correlative data that await prospective clinical validation.

Intratumoral heterogeneity and temporal dynamics, discussed in Section "Caveats: heterogeneity, spatial architecture, and temporal dynamics", further complicate any single-axis classification of resistant tumors; spatial and single-cell profiling of resistance-phase biopsies [131] will be essential to determine whether the three-axis framework accommodates this complexity or requires finer-grained models.

The next step is prospective validation. Paired pre-treatment and resistance-phase biopsies, longitudinal circulating tumor DNA (ctDNA) profiling, and spatially resolved immune monitoring will be needed to determine whether resistant tumors can be classified reproducibly by dominant visibility, access, or effector-function deficits. A practical near-term step would be retrospective application of this classification to existing cohorts with paired baseline and resistance-phase biopsies plus immune correlative data. Clinically, such work could support adaptive trial designs in which detection of a resistance state triggers mechanism-matched combination therapy rather than empiric escalation alone, ideally before clinical progression becomes fully consolidated.

## Data Availability

No datasets were generated or analysed during the current study.

## Abbreviations

ADC: Antibody-drug conjugate
AI: Artificial intelligence
AR: Androgen receptor
AR-V7: Androgen receptor splice variant 7
B2M: β2-microglobulin
BRCA: Breast cancer susceptibility gene
CAF: Cancer-associated fibroblast
CAR-T: Chimeric antigen receptor T-cell
CDK4/6: Cyclin-dependent kinase 4/6
cGAS-STING: Cyclic GMP-AMP synthase-stimulator of interferon genes
CRPC: Castration-resistant prostate cancer
CSF1R: Colony-stimulating factor 1 receptor
ctDNA: Circulating tumor DNA
CTLA-4: Cytotoxic T-lymphocyte-associated protein 4
CXCR2: C-X-C motif chemokine receptor 2
dMMR: Mismatch repair-deficient
DNMT: DNA methyltransferase
EGFR: Epidermal growth factor receptor
EMT: Epithelial-mesenchymal transition
ER: Estrogen receptor
ESR1: Estrogen receptor 1
GDF-15: Growth differentiation factor 15
HDAC: Histone deacetylase
HER2: Human epidermal growth factor receptor 2
HEV: High endothelial venule
HLA: Human leukocyte antigen
HR: Homologous recombination
IFN: Interferon
IL-6: Interleukin-6
JAK1/2: Janus kinase 1 and 2
KEAP1: Kelch-like ECH-associated protein 1
KRAS: Kirsten rat sarcoma viral oncogene homolog
LAG-3: Lymphocyte activation gene 3
LOH: Loss of heterozygosity
MDSC: Myeloid-derived suppressor cell
MET: MET proto-oncogene receptor tyrosine kinase
MHC: Major histocompatibility complex
MSI-H: Microsatellite instability-high
NK: Natural killer
NSCLC: Non-small-cell lung cancer
PARP: Poly(ADP-ribose) polymerase
PARPi: PARP inhibitor
PD-1: Programmed cell death protein 1
PD-L1: Programmed death-ligand 1
pHLA: Peptide-HLA complex
STING: Stimulator of interferon genes
STK11: Serine/threonine kinase 11
T-DXd: Trastuzumab deruxtecan
TAM: Tumor-associated macrophage
TCE: T-cell engager
TCR: T-cell receptor
TGF-β: Transforming growth factor-β
TIGIT: T-cell immunoreceptor with Ig and ITIM domains
TKI: Tyrosine kinase inhibitor
TLS: Tertiary lymphoid structure
TME: Tumor microenvironment
VEGF: Vascular endothelial growth factor
VEGFR2: Vascular endothelial growth factor receptor 2
VISTA: V-domain Ig suppressor of T-cell activation
